# Virtual karyotyping with SNP microarrays reduces uncertainty in the diagnosis of renal epithelial tumors

**DOI:** 10.1186/1746-1596-3-44

**Published:** 2008-11-06

**Authors:** Jill M Hagenkord, Anil V Parwani, Maureen A Lyons-Weiler, Karla Alvarez, Robert Amato, Zoran Gatalica, Jose M Gonzalez-Berjon, Leif Peterson, Rajiv Dhir, Federico A Monzon

**Affiliations:** 1 Department of Pathology, University of Pittsburgh, Pittsburgh, PA, USA; 2 Clinical Genomics Facility, University of Pittsburgh, Pittsburgh, PA, USA; 3 Department of Pathology, The Methodist Hospital Research Institute, Houston, TX, USA; 4 Department of Pathology, Creighton University Medical Center, Omaha, NE, USA; 5 Department of Pathology, The Methodist Hospital, Houston, TX, USA; 6 Pathology, Weill Medical College of Cornell University, New York, NY, USA

## Abstract

**Background:**

Renal epithelial tumors are morphologically, biologically, and clinically heterogeneous. Different morphologic subtypes require specific management due to markedly different prognosis and response to therapy. Each common subtype has characteristic chromosomal gains and losses, including some with prognostic value. However, copy number information has not been readily accessible for clinical purposes and thus has not been routinely used in the diagnostic evaluation of these tumors. This information can be useful for classification of tumors with complex or challenging morphology. 'Virtual karyotypes' generated using SNP arrays can readily detect characteristic chromosomal lesions in paraffin embedded renal tumors and can be used to correctly categorize the common subtypes with performance characteristics that are amenable for routine clinical use.

**Methods:**

To investigate the use of virtual karyotypes for diagnostically challenging renal epithelial tumors, we evaluated 25 archived renal neoplasms where sub-classification could not be definitively rendered based on morphology and other ancillary studies. We generated virtual karyotypes with the Affymetrix 10 K 2.0 mapping array platform and identified the presence of genomic lesions across all 22 autosomes.

**Results:**

In 91% of challenging cases the virtual karyotype unambiguously detected the presence or absence of chromosomal aberrations characteristic of one of the common subtypes of renal epithelial tumors, while immunohistochemistry and fluorescent in situ hybridization had no or limited utility in the diagnosis of these tumors.

**Conclusion:**

These results show that virtual karyotypes generated by SNP arrays can be used as a practical ancillary study for the classification of renal epithelial tumors with complex or ambiguous morphology.

## Background

Each year in the United States, there are approximately 31,900 cases of kidney and upper urinary tract cancer that account for approximately 3% of adult malignancies, and result in more than 11,900 deaths [1]. The most common types of renal epithelial tumors are clear cell renal cell carcinoma (RCC) (75%), papillary RCC (10%), chromophobe RCC (5%), and benign oncocytoma (5%)[2]. Correct pathological classification is critical for prognosis, management, therapy, and eligibility for clinical trials. Classification of renal epithelial tumors is primarily based on cytologic appearance, cell type of origin, and growth pattern. Morphological distinction among the renal cell tumor subtypes is generally straightforward after routine pathologic examination of the tissue. However, a subset of cases are ultimately diagnosed as renal cell carcinoma, unclassified, due to the presence of non-specific features that can be seen in all of the subtypes (such as granular, oncocytic or sarcomatoid morphology) [3] or the presence of more than one subtype in the same tumor [2].

Specific genetic abnormalities have been found in the different types of renal cell tumors and have been well characterized in the literature [4]. More than 98% of clear cell RCC show deletions in the p arm of chromosome 3, while papillary RCC usually presents with trisomies of chromosomes 7 & 17 and/or loss of chromosome Y. Chromophobe RCC is characterized by a hypodiploid chromosomal complement with monosomies of chromosomes 1, 2, 6, 10, 13, 17 and 21. Oncocytoma shows either normal chromosomes or copy number alterations in chromosome 1. Although the chromosomal alterations that characterize each subtype of renal epithelial neoplasms have been known for some time, this knowledge has not been routinely utilized in the diagnostic evaluation of these tumors. More than a decade ago, Steiner and Sidransky proposed the use of loss of heterozygosity (LOH) analysis with microsatellites to detect specific chromosomal deletions in renal tumors [5]. However, these assays have not been incorporated into clinical practice. Metaphase karyotyping can be used to detect these characteristic chromosomal lesions but it is not routinely used for diagnosis of renal tumors because fresh tissue is typically not available and solid tumors often grow poorly in culture. In addition karyotypes from solid tumors are especially challenging for cytogeneticists due to complex rearrangements and less than optimal banding patterns. Given the clinical relevance of accurately classifying patients for prognostic implications and therapeutic decisions, there is a need for diagnostic tools that reliably detect and quantify genetic lesions that are diagnostic for each subgroup of renal epithelial neoplasms.

Several techniques have been utilized for genome-wide scanning of chromosomal aberrations in renal tumors, including comparative genomic hybridization (CGH)[6], array CGH [7], and SNP arrays [8]. Array CGH has been used to accurately classify RCCs by histologic type, based on the specific genetic alterations described above [7,9]. However, most studies have been descriptive and have not attempted to develop and validate a molecular diagnostic tool for chromosome copy number analysis to classify diagnostically challenging tumors. In addition, a limitation of array CGH is that it cannot detect regions of 'copy neutral LOH' or acquired uniparental disomy (UPD) which has been reported to constitute 50–80% of the LOH in human cancers [10-13]. SNP arrays been used successfully to detect structural variations in several types of cancer, including RCC [8,11,14-19]. These arrays are similar to array CGH in that fragmented genomic DNA is amplified and applied to an array with the chromosomes reassembled *in silico*. However, instead of cDNA or BAC probes, SNP arrays use synthesized oligonucleotide probes optimized to identify specific alleles at each SNP locus. Thus, in addition to copy number information, SNP arrays also provide genotypes which can be used to determine regions of LOH. Other advantages include the ability to use either fresh or paraffin-embedded tissues [20-22], relatively low cost, good manufacturing practices, and that they are amenable to scalability and automation. Combined genome-wide copy number and LOH analysis with SNP arrays has also been referred to as "molecular allelokaryotyping" [23] and SOMA (SNP oligonucleotide microarray analysis) [24]. The potential clinical applications for SNP microarrays in molecular oncology assays are evident and we consider that this platform is suitable for diagnostic/prognostic test development for chromosome copy number analysis of human tumors.

The purpose of this study was to assess the utility of SNP array virtual karyotypes in the diagnosis of renal epithelial tumors that are not readily classifiable by standard histopathologic evaluation. Our previous work has shown that characteristic genetic lesions are readily identifiable in morphologically classic renal epithelial tumors [8]. Here, we extend the classic morphology cohort (n = 50) as well as assess the performance of the SNP array virtual karyotype on 25 morphologically challenging renal epithelial tumors. Our results indicate that virtual karyotypes can be used to reduce uncertainty in the classification of renal tumors and may be a useful ancillary study in clinical practice.

## Materials and methods

### Tissue samples

Fifty tumors with classic morphology from the four most common renal cell tumor subtypes (Clear Cell n = 21, Papillary n = 9, Chromophobe n = 9 and Oncocytoma n = 11) were used as a reference cohort to establish common profiles for each group (Classic Morphology Cohort). Reference samples were obtained from the pathology archives of the University of Pittsburgh Medical Center (UPMC, Pittsburgh, PA), The Methodist Hospital (TMH, Houston, TX), and Creighton University Medical Center (CUMC, Omaha, NE). In addition, twenty-five (25) morphologically challenging (MC) renal epithelial tumors were obtained from the Health Sciences Tissue Bank of the University of Pittsburgh. Samples were considered morphologically challenging if they fit into the following 3 groups: A) a definitive diagnosis was rendered but uncertainty in the diagnosis was expressed in the pathology report (n = 12), B) a definitive diagnosis could not be rendered after routine pathology examination but a favored (or consistent with) diagnosis was indicated (n = 6) or C) a diagnosis of an unclassified renal neoplasm was rendered (n = 7). Samples were de-identified and obtained under IRB approved protocols. All cases were reviewed by at least two pathologists and specifically the challenging cases were reviewed by genitourinary pathologists to confirm their status as diagnostically challenging. Ten 10 μm slides were obtained for all samples with a corresponding H&E stained slide. H&E stained tissue slides were evaluated by a pathologist (FAM or JMH) for selection of areas to be analyzed. Twenty of the samples with classic morphology and 3 of the morphologically challenging cases were described in a previous manuscript [8]. A summary of the samples used in this study is presented in Table 1. A complete list of samples belonging to the morphologically "classic" and morphologically "challenging" cohorts is available as Additional File 1.

**Table 1:** Summary of tumor samples (n = 75)

**Diagnosis**	**Number of Samples**	**Cohort (Morphology)**	**T Stage (n)**
Oncocytoma	11	Classic	N/A

Renal Cell Carcinoma, Chromophobe	9	Classic	T1b (2)
			T2 (4)
			T3a (2)
			T3b (1)

Renal Cell Carcinoma, Clear Cell	21	Classic	T1a (4)
			T1b (7)
			T2 (3)
			T3a (4)
			T3b (1)
			T4 (1)

Renal Cell Carcinoma, Papillary Type 1	5	Classic	T1b (2)
			T2 (1)
			T3b (1)

Renal Cell Carcinoma, Papillary Type 2	4	Classic	T1a (1)
			T1b (1)
			T3b (2)

Oncocytic tumors, favor oncocytoma	5	Challenging	N/A

Oncocytic/Eosinophilic tumors, favor carcinoma	10	Challenging	T1a (3)
			T1b (2)
			T2 (2)
			T3a (3)

Oncocytic/Granular tumors, favor chromophobe carcinoma	3	Challenging	T1a (1)
			T1b (2)

RCC, Unclassified	4	Challenging	T2 (1)
			T3a (2)

Mucinous, Tubular and Spindle cell carcinoma	1	Challenging	T1b (1)

Papillary RCC	2	Challenging	T1a (2)

*Note: A full list of all classic and challenging tumors in the study is available as Additional Table 1

### Sample preparation and extraction

Tumor DNA was obtained from manually microdissected 10 μm paraffin sections according to a previously described protocol for deparaffinization and DNA extraction [21]. DNA was quantitated on a NanoDrop ND-1000 spectrophotometer (NanoDrop Technologies, Wilmington, DE). All samples processed for downstream analysis in this study had an OD 260/280 ratio higher than 1.8.

### SNP array assay

Samples were processed with an FFPE-optimized protocol based on the GeneChip Mapping 10 K Xba Assay Kit (Affymetrix, Santa Clara, CA) and whose performance has been described previously [21]. All samples with 12 to 20 μg of PCR product were fragmented and labeled according to the standard Affymetrix Genotyping protocol. The samples were then hybridized on GeneChip^® ^Mapping 10 K 2.0 arrays (Affymetrix, Santa Clara, CA), for 16 h at 48°C in a GeneChip^® ^450 hybridization oven (Affymetrix, Santa Clara, CA) at 60 rpm. The arrays were washed and stained according to the Affymetrix Genotyping protocol. The SNP array data discussed in this manuscript have been deposited in NCBI's Gene Expression Omnibus (GEO ) and are accessible through GEO series accession numbers GSE9469.

### Data analysis

Data acquired from the Affymetrix GeneChip Operating System v4.0 (GCOS) was analyzed using Affymetrix GeneChip Genotyping Analysis Software (GTYPE) 4.1. The quality control parameters evaluated for each sample were signal detection rate (the percentage of features in the array that show adequate fluorescence intensity) and the SNP call rate (rate of successful allele identification) [20]. Data from the X chromosome was not analyzed, as the samples were not gender-matched. LOH and copy number estimates were obtained using a publicly available analysis package, Copy Number Analyzer for Affymetrix GeneChip arrays (CNAG 3.0) [25,26], as described before [8]. All samples used in downstream analysis had SNP call rates >85%.

Three pathologists participated in the assignment of consensus performance scores for morphology, IHC, FISH, and SNP arrays (FAM, AVP, JMH). A test was considered 'diagnostic' if information provided by the ancillary study alone could enable a general surgical pathologist to categorize the tumor. The SNP-based virtual karyotype diagnosis was established by evaluating chromosomal aberrations and LOH in each sample from the CNAG output and determining the assignment to a tumor subtype with the following criteria: -3p, clear cell carcinoma; +7/+17, papillary carcinoma; -1, -2, -6, -17 (with or without -10 or -13), chromophobe carcinoma; and -1 or normal/diploid, oncocytoma. Chi-square contingency table analysis was performed using Stata (Version 10, College Station, TX) to determine if tumor size (cm), Fuhrman nuclear grade, organ confined (y/n), or low-high stage (I, II vs. III, IV) was associated with chromosomal lesions previously reported as having prognostic value.

### FISH

Interphase FISH studies on renal tumors were performed as part of the clinical diagnostic workup according to standard protocols and results were obtained through de-identified clinical records. The standard renal cell carcinoma (RCC) FISH panel at UPMC consists of centromeric probes for chromosomes 1, 2, 7, and 17. Formalin-fixed paraffin-embedded sections, were mounted, and serially sectioned at 5-mm intervals. H&E section was used by a pathologist to determine the area of the tissue to be targeted for analysis. FISH slides were deparaffinized in xylene twice for 10 minutes, dehydrated twice with 100% ethanol and then pretreated using the Vysis Paraffin Pretreatment Kit (Vysis, Inc., Downers Grove, IL). Slides were digested for 18 minutes in protease solution (0.5 mg/ml) at 37°C. FISH was performed using CEP1, CEP2, CEP7 and CEP17 centromere probes (Vysis, Inc.). The target slide was denatured at 75°C for 5 minutes and dehydrated in 70%, 85%, and 100% ethanol. Slides were incubated with probe overnight at 42°C in a humidified chamber. Post-hybridization washes were performed using 0.4× SSC/0.3% Igepal (Sigma) at 72°C for 2 minutes, followed by a room temperature 2 × SSC/0.1% Igepal wash for 30 seconds. Slides were air-dried in the dark and counterstained with DAPI (Vysis, Inc). Analysis was performed using a Nikon Optiphot-2 (Nikon, Inc) and Quips Genetic Workstation equipped with Chroma Technology 83000 filter set with single band excitors for Texas Red/Rhodamine, FITC, DAPI (uv 360 nm) (Vysis, Inc). Only individual and well delineated cells were scored. Overlapping cells were excluded from the analysis. Approximately 60 cells were analyzed in the targeted region. By standard clinical interpretative guidelines, chromosomal losses are considered significant if present in greater than 30% of cells. The loss is indeterminate if present in 20–30% of cells. The loss is considered artifactual if seen in less than 20% of cells. Chromosomal gains are considered significant if present in greater than 20% of cells. The gain is considered artifactual if seen in less than 20% of cells. If multiple sections with different morphology were subjected to FISH analysis for the same sample, the results were averaged for each probe. The FISH-based diagnosis was established by evaluating chromosomal losses in each sample and determining the assignment with the following criteria: -1 only or normal/diploid, oncocytoma; -1, -2, and -17, chromophobe carcinoma; +7 and/or +17 papillary carcinoma. Cases with results not fitting any of these groups were deemed non-diagnostic.

## Results

### Classic morphology cohort

**Figure 1:**
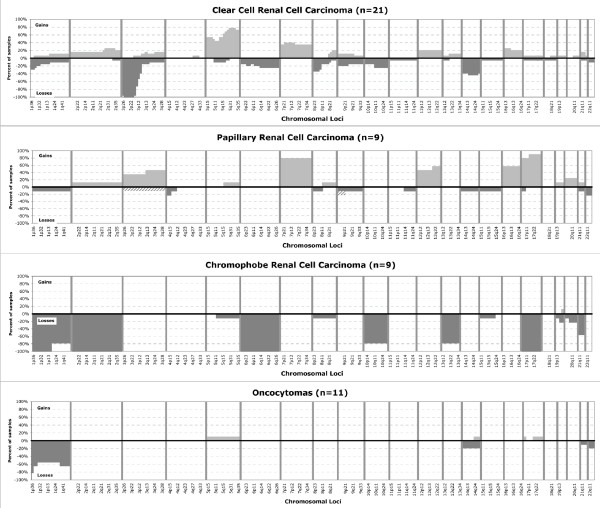
**Cumulative frequency of chromosomal lesions in the four most common subtypes of renal epithelial tumors**. Gains are indicated as positive values (light gray) and losses as negative values (dark gray). Hashed bars indicate lesions identified as copy neutral LOH.

The chromosomal copy number aberrations and loci of LOH derived using the SNP arrays were in agreement with those reported in the literature for each subtype. Figure 1 summarizes the lesions detected in the 50 morphologically classic renal tumors in our study. Loss of all or part of the short arm of chromosome 3 is a distinctive feature of clear cell tumors, and was detected in all samples from this group by the SNP arrays. The minimum region of loss detected was from 3p21-p25. Seven out of eight papillary carcinoma samples demonstrated the characteristic trisomies of chromosomes 7 and 17, while one papillary tumor failed to show these aberrations and showed other chromosomal changes, which have been reported previously for type 2 renal papillary carcinoma [27]. The chromophobe carcinoma cohort showed losses affecting chromosomes 1, 2, 6 and 17 in all tumors, in addition, losses of 10 and 13 were observed in 8 of 9 tumors. Oncocytomas showed complete or partial loss of chromosome 1 in 82% of these tumors and no chromosomal copy number imbalances in 18%. Thus, tumors in this reference cohort show the characteristic virtual karyotype profiles that concur with morphology and permit classification into each of the four most common diagnostic categories of renal cell neoplasms in 49/50 cases (98%).

In addition to the characteristic changes reported, the SNP arrays identified additional copy number aberrations. Clear cell carcinomas showed trisomy 5 in 52% of cases and gain of 5q in 76%, which makes +5q the second most common chromosomal lesion in this tumor subtype. Interestingly, trisomy 7, a chromosomal lesion mostly associated with papillary RCC, was seen in 33% of clear cell carcinomas. Chromosomal lesions previously reported as associated with poor outcome in clear cell tumors were identified in 43% (-14q) and 19% of patients (-9q) [9,28] and only two cases showed concurrent loss of 14q and 9p. In this cohort, the presence of these lesions was not associated with advanced stage (Stage I/II vs. III/IV), higher nuclear grade or presence of metastasis (P > 0.05 with Fisher's exact test). Papillary RCC showed frequent gains of chromosome 3 (+3q in 44%), trisomy or partial gain of chromosome 12 (+12q in 56%) and trisomy 16 in 56% of cases. In chromophobe carcinomas, loss of 21q was the most common lesion (56%) apart from those that define this group. In oncocytomas, lesions in 14q and chromosome 22 were observed, albeit at low frequencies. A list of all chromosomal lesions seen in the morphologically classic tumors summarized by chromosomal arm is available as Additional File 2.

### Identification and implications of tumor subtype in the morphologically challenging cohort

In the morphologically challenging cohort, 22 out of 25 cases (88%) passed the quality thresholds for analysis as described in methods. Repeat analysis of these three samples was not attempted. For the 22 cases used in the final analysis, SNP arrays were able to identify genomic gain/loss patterns that matched one of the classic patterns identified in the reference cohort in 86% (19 of 22). One of the cases with non-classic genomic patterns matched the published genomic profile for mucinous tubular and spindle cell carcinoma (MTSCC) reported by Rokozy, *et al *(monosomies in chromosomes 1, 4, 6, 8, 9, 13, 14, 15, 22) [29]. Thus, in 91% of these challenging cases with adequate array results, a genomic pattern characteristic of a known tumor type was identified (Table 2). MC09 could not be classified due to showing a novel genomic profile with mixed features for clear cell and chromophobe carcinomas. The second case that could not be classified (MC12) showed a novel genomic profile with some features of papillary renal carcinoma (+12, +16) with additional lesions not seen in other papillary tumors in the classic morphology cohort (+5q, +9p with UPD, -17p, del11q). Taking into account all cases (including those that failed array quality thresholds) the virtual karyotype was able to classify 80% of cases tested in the morphologically challenging cohort. Comparatively, FISH failed to reliably detect classic genomic loss patterns in this cohort (0/19) (Table 2). Screen shots of the actual virtual karyotypes with the associated FISH data for each of the morphologically challenging tumors are available as Additional File 3.

**Table 2:** Performance of Molecular Ancillary Studies for Diagnosis of Renal Cell Tumors

**Cohort (n)**	**Morphology Diagnostic**	**SNP Diagnostic**		FISH Diagnostic*	
Classic (50)	50	49/50	98%	3/18	17%
Challenging (25)	0	20/22	91%	0/19	0%

*Total (75)*	50	69/72	96%	3/37	8%

*Not all samples have clinical data for FISH.

**Figure 2:**
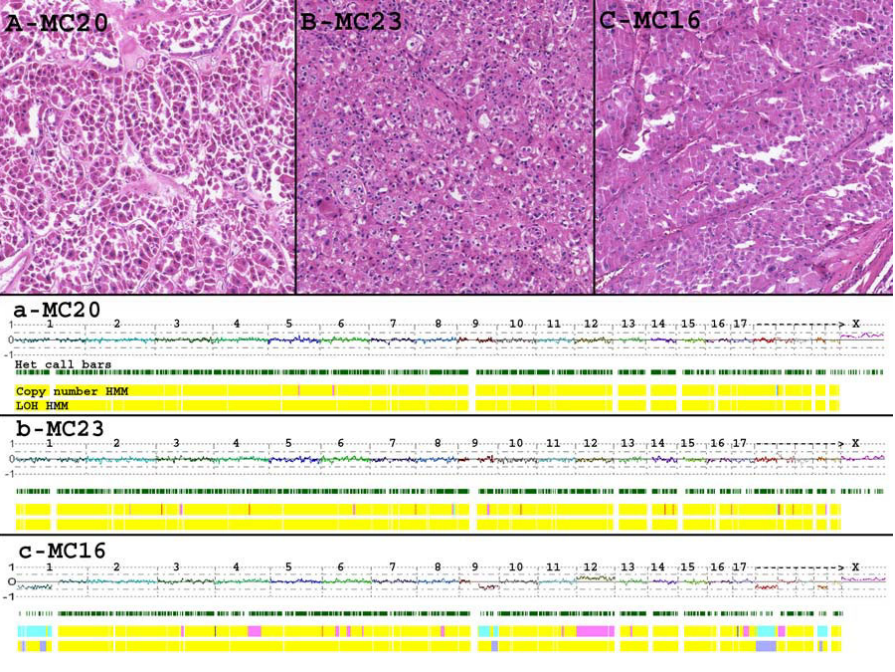
**Representative tumors from the morphologically challenging cohort**. **A, B & C**: Photomicrographs of oncocytic tumors (100×). MC20 and MC23 with diagnosis of eosinophilic renal cell carcinomas; MC16 diagnosed as oncocytic renal cell neoplasm, favor chromophobe carcinoma. **a, b and c**: whole genome view of virtual karyotypes of samples A, B and C respectively. The virtual karyotypes for these tumors show chromosomal patterns consistent with those seen in oncocytomas. The uppermost plot for each sample represents the estimated copy number as a log 2 ratio averaged over 20 SNPs, color-coded Hidden Markov Model (HMM) for copy number (yellow = copy number 2, pink = copy number 3, aqua = copy number 1), color-coded HMM for LOH (yellow = no LOH, blue = LOH).

All tumors in the morphologically challenging cohort were, by definition, non-straightforward cases that required ancillary studies and consultation with other genitourinary pathologists. The final diagnoses ranged from a qualified categorization (e.g., "most consistent with" the diagnosis of x) to completely unclassifiable. Often times the morphology, FISH, and IHC results suggested conflicting diagnoses, which affected the certainty of the diagnosis reported. Table 3 summarizes the results of the virtual karyotype for each morphologically challenging tumor and gives the associated FISH results and surgical pathology diagnosis. The potential impact of the virtual karyotype, had it been available, is estimated by determining whether the results confirmed the pathologists' favored diagnosis (confirms), provides results that are discrepant with the final diagnosis (discrepant), were able to classify the tumor when it could not be classified by standard pathologic criteria (SNP diagnostic), or identified a clear, but "novel" pattern that was not seen in the morphologically classic cohort (novel). The diagnostic implications of the virtual karyotypes in the 22 morphologically challenging tumors is summarized in Table 4. There were 13 virtual karyotypes that supported the diagnosis that the pathologist favored despite the implicit uncertainty. He or she may have been able to provide a more confident final diagnosis had they had the results of the virtual karyotype. There were 4 cases that were signed out as a carcinoma (malignant) that have virtual karyotypes consistent with a benign oncocytoma. There were no cases signed out as a benign oncocytoma that had karyotypes consistent with malignant types. Lastly, there were 3 cases that were signed out as unclassifiable tumors which the SNP array virtual karyotype could classify the tumor. This classification would have given the surgeons and oncologists a direction for treatment and follow up of these patients had this assay been available. Figure 2 shows examples of tumors that were interpreted as malignant (eosinophilic or unclassified carcinomas) but whose virtual karyotypes suggest that they are oncocytomas.

**Table 3:** FISH panel and virtual karyotype results for 22 morphologically challenging renal tumors

**Sample ID**	**Histologic Diagnosis**	**FISH**	**Virtual Karyotype**	**Virtual Karyotype Interpretation**	Array Result vs. Final Diagnosis*	**Outcome**	**Months Follow up**
MC01	Low grade neoplasm, favor oncocytoma	Not done	-1, -14, -21	Oncocytoma	Confirms	No evidence of tumor	9

MC02	Low grade neoplasm, favor oncocytoma	Not done	del(10)(p11.23-p14)	Oncocytoma	Confirms	Disease status Unknown	1

MC09	Eosinophilic epithelial tumor morphologically consistent with eosinophilic renal cell carcinoma	-1 (44%), -2 (52%), -7 (38%), and -17 (88%)	-1, -1, -2, -3, +5, UPD 6, +7, -9, -9, -10p, -10p, del(10)(q24.33-qter), -11, -11, +12, -13,-13, +15(q22.2-qter), +16p, -17p, -17p, -17p, -17q, -17q, -18, +19, -21, -21, -22, -22, -22 | [inferred tetrasomy]	Mixed pattern CRCC/CHRCC = Unclassified	Novel pattern	Local & regional lymph node recurrence 52 months after nephrectomy Alive at last F/U	69

MC10	Oncocytic renal neoplasm, favor carcinoma	-1 (51%), 2 failed, +7 (21%), +7+7 (8%), +17 (30%) and intermediate -17(28%)	-1, -14	Oncocytoma	SNP diagnostic	No evidence of tumor	30

MC11	Renal cell carcinoma, clear cell type with focal granular (eosinophilic) morphology	CHRCC area: +2 (75%) with intermed -1, -7, -17; CRCC area: -1, -2, -17 (34%, 36%, 30%)	UPD(3)(p14.1-p13.2)	Clear Cell RCC	Confirms	No evidence of tumor	33

MC12	Eosinophilic variant of clear cell renal carcinoma with papillary features.	-2 (31%), -7 (37%), -17 (97%)	del(4)(p15.1-pter), +5(q21.3-qter), +9p(UPD), del(11)(q13.3-qter), +12, +16, del(17p), +17(q21.32-qter), +17(q21.32-qter), +20, -22	Novel, not consistent with clear cell	Novel pattern	Never disease free. Deceased	2

MC13	Oncocytic renal cell carcinoma, most suggestive of eosinophilic variant of conventional clear cell carcinoma	-1 (97%), -2 (83%), -7 (70%), and -17 (97%)	UPD(3)(p12.2-p24.1)	Clear Cell RCC	Confirms	No evidence of tumor	3

MC14	Chromophobe renal cell carcinoma	-1 (48%), -2 (50%), -7 (56%), and -17 (47%)	No detectable chromosomal abnormalities	Oncocytoma	Discrepant	No evidence of tumor	29

MC15	Renal cell carcinoma with morphologic features consistent with eosinophilic variant of clear cell carcinoma	-2 (37%), -7 (45%), -17 intermediate (20%)	del(1)(p32.3-pter), del(3)(p12.2-pter)	Clear Cell RCC	Confirms	Never disease free. Deceased	19

MC16	Oncocytic renal epithelial neoplasm, favor chromophobe renal cell carcinoma with eosinophilic morphology	-1(48%), -2 (40%), -7 (36%), and -17 (42%)	-1p, -9q, +12, -18, -21	Oncocytoma (novel)	Discrepant	No evidence of tumor	30

MC18	Low grade carcinoma with myxoid matrix and spindle and tubular architecture	-1 (85%), -2 (67%), -7 (37%), and -17 (73%)	-1, -4, -6, -8, -9, -13, -14, -15, -17, -22	MTSCC	Confirms	No evidence of tumor	19

MC19	Renal cell carcinoma with morphologic features of a chromophobe renal cell carcinoma. Multiple other tumors (2 papillary, 2 clear cell)	-1 (38%), -2 (42%), -7 (33%). 17 failed.	-3p, +3q, +7, del(9)(p13.2-p22.3)	Clear Cell RCC	Confirms	No evidence of tumor	21

MC20	Eosinophilic renal cell carcinoma	Intermed -1 (25%), -2 (52%), -7 (42%), -17	No detectable chromosomal abnormalities	Oncocytoma	Discrepant	No evidence of tumor	24

MC21	Oncocytic renal epithelial neoplasm	-1 (92%), -17 (37%), +7 (51%)	-1,+7, +9p, -9q	Oncocytoma (novel)	SNP diagnostic	No evidence of tumor	20

MC22	Renal cell carcinoma, unclassified	-1 (45%), 12 (37%), -17 (50%); intermed -7 (22%)	+7, +16	Papillary RCC	SNP diagnostic	No evidence of tumor	20

MC23	Eosinophilic renal cell carcinoma	-1 (49%), -2 (53%), -7 (49%), and -17 (74%)	No detectable chromosomal abnormalities	Oncocytoma	Discrepant	No evidence of tumor	22

MC24	Papillary renal cell carcinoma, type 2	-1 (48%), intermed -2 (23%), -7 (26%)	+7, +12, +17	Papillary RCC	Confirms	No evidence of tumor	27

MC26	Renal clear cell carcinoma	-2 intermed (23%). +7 (52%), -17 (43%)	-3p, unable to interpret other changes	Clear Cell RCC	Confirms	No evidence of tumor	1

MC28	Renal oncocytoma	-1 (37%), -2 (38%), -7 (30%), and -17 (55%)	No detectable chromosomal abnormalities	Oncocytoma	Confirms	No evidence of tumor	68

MC29	Papillary renal cell carcinoma, type II	-2 intermediate (20%),+7 (30%), +7+7 (18%)	+7, +16q (high normal contamination)	Papillary RCC	Confirms	No evidence of tumor	16

MC30	Renal oncocytoma	1 -(41%), 2 failed, -7(34%), -17(65%)	No detectable chromosomal abnormalities	Oncocytoma	Confirms	No evidence of tumor	71

MC31	Renal epithelial oncocytic neoplasm with features of an oncocytoma	-1 (67%), -2 (63%), -7 (42%), and -17 (40%)	No detectable chromosomal abnormalities	Oncocytoma	Confirms	No evidence of tumor	19

*Array Results vs. Final Diagnosis field summarizes the potential impact on diagnosis had the SNP array karyotype been available (confirms = virtual karyotype confirms the favored diagnosis; SNP diagnostic = the virtual karyotype could classify the tumor based on the pattern of genetic lesions while morphology with IHC and FISH could not; novel = new pattern seen on virtual karyotype and ambiguous morphology; discrepant = virtual karyotype diagnosis and morphologic diagnosis are discrepant).

**Table 4:** Diagnostic Implications of SNP Array Results in Morphologically Challenging Cohort*

Confirms	Diagnosis* confirmed by SNP	13
Discrepant	Diagnosis carcinoma (malignant), SNP OC (benign)	4
Discrepant	Diagnosis OC, SNP carcinoma	0
SNP diagnostic	RCC Unclassified**, SNP diagnostic	3

*Tumors were classified, but with uncertainty due to complex morphology and/or conflicting ancillary study results

**Tumors could not be classified by morphology and ancillary studies.

In two cases where the SNP arrays suggested a diagnosis of oncocytoma, the tumors showed chromosomal lesions not observed in our reference (classic morphology) cohort. These changes include +7, +9p, -9q, +12, and -18. Chromosomal gains are rare in oncocytomas, but gains of 7, 14, 18 and 20 have been previously reported [30,31]. Gain of 9p with concurrent loss of 9q in one of these tumors suggests the presence of an isochromosome 9p, which has also been reported in oncocytomas [30]. Gain of 12 and loss of 18 have not, to our knowledge been previously reported. Trisomy 7 and 12 have been reported in the setting of familial oncocytomas, however, most familial tumors show no chromosomal abnormalities [32]. Interestingly, these two oncocytomas from our study with more complex chromosomal patterns presented with atypical clinical features. One of them, MC21, showed invasion of the perirenal adipose tissue and was diagnosed as an unclassifiable renal epithelial neoplasm. The other tumor, MC16, is from a patient who was diagnosed with an ipsilateral oncocytoma 8 years earlier. Tumor MC16 was diagnosed as an oncocytic renal tumor with a differential diagnosis of chromophobe carcinoma versus clear cell carcinoma. The virtual karyotype is not consistent with either chromophobe carcinoma (1, 2, 6, 10, 13, and 17) or a clear cell carcinoma (-3p, +5q), but rather is most consistent with genetic lesions seen in oncocytomas as described above (Figure 2c). It is unclear if these chromosomal lesions are associated with a more "aggressive" behavior in oncocytomas, however, at last follow-up (21 and 30 months after surgery), both patients were alive and without evidence of tumor.

## Discussion

Specific chromosomal abnormalities have been found in different types of renal epithelial neoplasms that define the four most common subtypes of renal tumors (clear cell RCC, papillary RCC, chromophobe RCC, and oncocytoma) [4,9,33] and other less common subtypes such as the mucinous, tubular and spindle cell carcinoma (MTSCC) [29]. We and others have shown that these abnormalities can be detected with array-based whole genome copy number analysis [7-9], and that reliable detection of copy number abnormalities in paraffin embedded tissues can be performed with SNP genotyping arrays without the need for patient-matched normal tissue [21,22,25].

In addition to performing well on formalin-fixed paraffin embedded tumors, a useful ancillary study for classification of renal tumors needs to perform well on morphologically challenging tumors, not just classic tumors which can be reliably diagnosed by routine pathologic examination of H&E stained slides. To address this issue, we analyzed SNP array virtual karyotypes for 25 renal epithelial tumors that could not be readily categorized by standard pathology review and were confirmed to be 'morphologically challenging' by two independent genitourinary pathologists. The diagnoses for these cases conveyed a degree of uncertainty in the diagnosis, even when a tumor subtype was provided. Some tumors could simply not be categorized and were signed out as unclassifiable renal tumors. SNP array virtual karyotypes were able to categorize 91% of these challenging tumors whose DNA was suitable for analysis (Table 2). The results of these virtual karyotypes could have impacted patient management had they been available as an ancillary study at the time these tumors were diagnosed (Table 4).

As indicated in this study and reported by others, ancillary studies currently used by pathologists, such as IHC and FISH, while reliable when evaluating renal tumors with classic morphology, often fail to unequivocally categorize morphologically challenging renal tumors [34,35]. Interphase FISH on paraffin-embedded tissues is known to be technically demanding, time-consuming, and requires complex protocols with difficult optimizations [36]. In addition, the protocol and the interpretation of the results are limited by the presence of necrosis and/or the sectioning of nuclei. It has been reported that FISH on 5 um sections of paraffin embedded tissue can underestimate chromosomal anomalies when compared to analysis of entire nuclei (37, 38). This artifact may give an underestimation of trisomies and overestimation of monosomies if the dimensions of the analyzable nuclei are not correctly evaluated [36]. In our study, when comparing to the SNP array results, interphase FISH on paraffin embedded renal tumors overestimated monosomies even with a conservative threshold (>30% of examined cell nuclei). Other authors have reported better results for FISH when background signals in the normal kidney tissue are substracted from the tumor [28]. Utilizing this approach could improve the performance of FISH for resolving morphologically challenging tumors.

Immunohistochemistry appears to be a reliable method for identification of common variants but fails to resolve oncocytic neoplasms and other less common renal tumor subtypes [34]. By definition, the tumors assigned to the morphologically challenging cohort could not be resolved based on morphology and routinely available immunohistochemistry stains. The fact that these tumors were investigated with IHC and still had some level of uncertainty in the diagnosis reflects the limitation of this technique in this type of tumors.

In summary, we believe that virtual karyotyping is a robust alternative to FISH and IHC for these types of diagnostic specimens. Even when considering samples with poor quality DNA, we were able to identify diagnostic chromosomal profiles in 80% of all cases, including rare subtypes such as the above mentioned MTSCC. These data suggest that SNP array virtual karyotypes would be a useful ancillary study for increasing diagnostic certainty in the pathologic evaluation of renal epithelial tumors.

### Clinical significance of classification and prognosis

SNP array virtual karyotypes can reliably categorize renal epithelial tumors, but what is the clinical significance of correct categorization? Proper diagnosis is critical to patient management decisions, and the SNP array karyotypes can identify well-established genetic lesions that categorize each renal tumor into a subtype [8]. For example, loss of 3p indicates that the tumor is a clear cell renal carcinoma and all of the clear cell carcinomas in our study show a loss of 3p. It has been shown that on univariate analysis, different histologic subtypes of renal cell tumors have markedly different prognosis with 5-year disease specific survival of 100% for oncocytoma and chromophobe RCC, 86% for papillary RCC, 76% for clear cell RCC and 24% for unclassified RCC [37]. However, the significance of histologic type for prognosis has not been confirmed on multivariate analysis, where TNM stage, nuclear grade and necrosis are best predictors of poor outcome. However, most studies are limited by the low frequency of non clear-cell histologic types and thus might be underpowered to definitively evaluate the role of histologic subtype as a prognostic factor [38]. Recent evidence suggests that the histologic subtype can predict response to combined immunotherapy [39]. In a study by Herrmann *et al*, patients with papillary RCC showed no significant benefit from combined IL-2/Interferon/5FU, and thus it is suggested that specific treatments be evaluated on each histologic subtype separately [40]. Importantly, new therapeutic approaches are being evaluated in clinical trials with strict inclusion criteria that include restriction of histologic subtypes and thus this information has strong implications on the therapeutic options for patients with RCC [38,41]. Therefore, accurate classification of renal tumors is important for patient management, as the morphologic subtype of renal cell carcinoma has been shown to be of prognostic significance [37] and some subtypes require specific therapeutic management [39,40,42].

### The role of additional genetic lesions

In addition to the 'disease-defining lesions' that permit categorization of renal cell neoplasms, the SNP array karyotypes allow us to also see additional lesions in the genome of these tumors.

Loss of the p arm of chromosome 9 and other non-characteristic copy number alterations are quite frequent in renal tumors and some have been associated with poor prognosis (9q/14q deletions) or more favorable outcomes (5q31-ter gains) in clear cell RCC [9,28,43-45]. Recently, Brunelli and colleagues confirmed the association of 9p loss with lower 5-year cancer specific survival in clear cell renal cell carcinoma [28]. Interestingly, in out cohort, -14q was present in 62% (5/8) of stage III/IV patients while it was only seen in 30% (4/13) of organ confined tumors (stage I/II), although this association did not reach statistical significance. However, determining this association was not the goal of this study, and thus the sample size was underpowered to detect it. The importance of these additional genomic lesions for clinical decision making is evolving and may soon be desirable to assess in clinical samples. If the status of additional genetic lesions has clinical utility, then it may be desirable to obtain virtual karyotypes on all clear cell carcinomas, not just the morphologically challenging tumors. Detecting the constellation of genetic lesions present in an individual tumor may enable us to provide additional diagnostic, prognostic, and therapeutic information which can enable the transition to personalized medicine for renal tumors.

### Limitations of SNP array virtual karyotypes for diagnostic molecular oncology

Array-based copy number platforms, whether standard arrayCGH or SNP arrays, provide high-resolution, genome-wide assessment of tumor genomes. However, they do have limitations. Since array-based copy number platforms provide *relative *copy number assessment of the tumor genome, tetraploid genomes generate the same virtual karyotype as a diploid genome. Although certain features of the SNP array virtual karyotype can suggest tetraploidy, such as a region of chromosomal loss that is associated with partial maintenance of heterozygosity [46]; tetraploidy cannot be reliably discerned from a subclone and/or normal cell contamination. In addition, since array-based copy number platforms reconstruct the genome *in silico *from disrupted DNA, they cannot detect inversions or balanced translocations.

The potential impact of SNP array virtual karyotypes to diagnostic molecular pathology, particularly solid tumors, is far reaching. SNP array karyotypes show promise as reliable, objective and relatively inexpensive diagnostic tools to interrogate the many genome-level events occurring in neoplasia – useful even at relatively low densities as described in this study. Although we highlight application to renal epithelial tumors in this manuscript, the possible applications to other cancers using low and higher density SNP arrays is already being explored by us and other authors [23,47-49].

Significant issues remain unanswered at key decision points in the management of patients with renal neoplasms. Unclassified renal cell carcinoma is a clinically relevant problem the impacts patient prognosis and treatment decisions. In addition, the lack of reliable biomarkers that can predict RCC recurrence in clinically localized tumors or that can predict therapeutic response have substantial impact in mortality. We believe that these issues can benefit from a genomic approach to detect chromosomal abnormalities and that the SNP arrays can enable the clinical application of this approach on renal epithelial neoplasms.

## Competing interests

The authors declare that they have no competing interests.

## Authors' contributions

JMH participated in conceptualization and design, retrieval and pathology review of specimens, compiled clinical and laboratory data, extracted DNA, analyzed SNP array data and wrote manuscript. FAM provided overall oversight of the project, participated in conceptualization and design, contributed cases, analyzed SNP array data, reviewed clinical and laboratory data, provided funding and reviewed and edited manuscript (final approval). AVP participated in design, identified and contributed cases, reviewed all specimens for inclusion, reviewed and edited manuscript. MLW and KA performed DNA extraction and SNP array assays RA, ZG, RD and JMG contributed cases. LEP performed statistical analysis. RD contributed FISH data and outcome information.

## Supplementary Material

Additional file 1 All samples in study (n = 75) with pathology descriptors. Click here for file

Additional file 2 Frequency of chromosomal gain/loss identified with SNP arrays in renal cell tumors with classic morphology (n = 50). Click here for file

Additional file 3 Virtual karyotypes of morphologically challenging tumors with associated surgical pathology and FISH data. Click here for file
