# Supplementary material for: Virtual karyotyping with SNP microarrays reduces uncertainty in the diagnosis of renal epithelial tumors
Source: Diagn Pathol. 2008 Nov 6;3:44. doi: 10.1186/1746-1596-3-44 (PMC2588560; doi:10.1186/1746-1596-3-44)
Supplement: Additional file 1 — All samples in study (n = 75) with pathology descriptors. [file 1746-1596-3-44-S1.pdf]

| n | Sample ID | Diagnosis | Cohort (Morphology) | Size (cm) | Fuhrman Nuclear Grade | TNM Stage |
|---|---|---|---|---|---|---|
| 1 | CHRCC02 | Oncocytoma | Classic | 4.5 | N/A | N/A |
| 2 | CUMC01 | Oncocytoma | Classic | 3.5 | N/A | N/A |
| 3 | MONC01 | Oncocytoma | Classic | 10.0 | N/A | N/A |
| 4 | OC01 | Oncocytoma | Classic | 3.5 | N/A | N/A |
| 5 | ONC02 | Oncocytoma | Classic | 1.6 | N/A | N/A |
| 6 | ONC03 | Oncocytoma | Classic | 6.5 | N/A | N/A |
| 7 | OC1 | Oncocytoma | Classic | 2.8 | N/A | N/A |
| 8 | OC2 | Oncocytoma | Classic | 5.5 | N/A | N/A |
| 9 | OC4 | Oncocytoma | Classic | 4.0 | N/A | N/A |
| 10 | OC5 | Oncocytoma | Classic | 1.5 | N/A | N/A |
| 11 | OC6 | Oncocytoma | Classic | 21.0 | N/A | N/A |
| 12 | CHRCC03 | Renal Cell Carcinoma, Chromophobe | Classic | 6.8 | 3 | T3a N0 Mx |
| 13 | DES08 | Renal Cell Carcinoma, Chromophobe | Classic | 6.0 | 3 | T2 Nx Mx |
| 14 | MRCC01 | Renal Cell Carcinoma, Chromophobe | Classic | 16.5 | 4 | T2 N2 Mx |
| 15 | MRCC10 | Renal Cell Carcinoma, Chromophobe | Classic | 14.9 | 3 | T3a Nx Mx |
| 16 | CHRCC1 | Renal Cell Carcinoma, Chromophobe | Classic | 7.7 | 2 | T2NxMx |
| 17 | CHRCC2 | Renal Cell Carcinoma, Chromophobe | Classic | 9.7 | 3 | T2NxMx |
| 18 | CHRCC3 | Renal Cell Carcinoma, Chromophobe | Classic | 5.5 | 3 | T1NxMx |
| 19 | CHRCC4 | Renal Cell Carcinoma, Chromophobe | Classic | 7.0 | 3 | T3bNxMx |
| 20 | CHRCC5 | Renal Cell Carcinoma, Chromophobe | Classic | 6.0 | 2 | T1bNxMx |
| 21 | CRCC01 | Renal Cell Carcinoma, Clear Cell | Classic | 6.2 | 3 | T1b Nx Mx |
| 22 | CUMC03 | Renal Cell Carcinoma, Clear Cell | Classic | 8.0 | 2 | T3b Nx Mx |
| 23 | CUMC04 | Renal Cell Carcinoma, Clear Cell | Classic | 6.0 | 2 | T1b N0 Mx |
| 24 | CUMC05 | Renal Cell Carcinoma, Clear Cell | Classic | 7.0 | 4 | T3a Nx Mx |
| 25 | CUMC06 | Renal Cell Carcinoma, Clear Cell | Classic | 5.5 | 3 | T1b Nx Mx |
| 26 | CUMC07 | Renal Cell Carcinoma, Clear Cell | Classic | 6.5 | 2 | T3a Nx Mx |
| 27 | CUMC09 | Renal Cell Carcinoma, Clear Cell | Classic | 2.5 | 2 | T1a Nx Mx |
| 28 | DES03 | Renal Cell Carcinoma, Clear Cell | Classic | 8.5 | 3 | T2 Nx Mx |
| 29 | DES05 | Renal Cell Carcinoma, Clear Cell | Classic | 8.5 | 4 | T3a Nx Mx |
| 30 | MRCC02 | Renal Cell Carcinoma, Clear Cell | Classic | 2.3 | 2 | T1a Nx Mx |
| 31 | RAD08 | Renal Cell Carcinoma, Clear Cell | Classic | 11.8 | 4 | T3a N0 Mx |
| 32 | RAD14 | Renal Cell Carcinoma, Clear Cell | Classic | 14.0 | 2 | T2 Nx Mx |
| 33 | RAD20 | Renal Cell Carcinoma, Clear Cell | Classic | 8.3 | 2 | T2 N2 M1 |
| 34 | RAD22 | Renal Cell Carcinoma, Clear Cell | Classic | 4.0 | 4 | T1b Nx M1 |
| 35 | CRCC1 | Renal Cell Carcinoma, Clear Cell | Classic | 4.0 | 2 | T1aNxMx |
| 36 | CRCC2 | Renal Cell Carcinoma, Clear Cell | Classic | 6.0 | 2 | T1bNxMx |
| 37 | CRCC3 | Renal Cell Carcinoma, Clear Cell | Classic | 5.4 | 2 | T1bNxMx |
| 38 | CRCC4 | Renal Cell Carcinoma, Clear Cell | Classic | 4.5 | 3 | T1bNxMx |
| 39 | CRCC5 | Renal Cell Carcinoma, Clear Cell | Classic | 5.2 | 2 | T1bNxMx |
| 40 | CRCC6 | Renal Cell Carcinoma, Clear Cell | Classic | 2.5 | 2 | T1a Nx Mx |
| 41 | RAD10 | Renal Cell Carcinoma, Clear Cell (Metastasis) | Classic | N/A | 4 | Tx Nx M1 |
| 42 | PRCC02 | Renal Cell Carcinoma, Papillary Type 1 | Classic | 1.7 | 3 | T1a Nx Mx |
| 43 | PRCC9 | Renal Cell Carcinoma, Papillary Type 1 | Classic | 2.0 | 3 | T3b N2 Mx |
| 44 | PRCC1 | Renal Cell Carcinoma, Papillary Type 1 | Classic | 5.3 | 3 | T1bNxMx |
| 45 | PRCC5 | Renal Cell Carcinoma, Papillary Type 1 | Classic | 8.5 | 3 | T2NxMx |
| 46 | PRCC6 | Renal Cell Carcinoma, Papillary Type 1 | Classic | 4.5 | 2 | T1bNxMx |
| 47 | CUMC02 | Renal Cell Carcinoma, Papillary Type 2 | Classic | 7.1 | 3 | T3b Nx Mx |
| 48 | MRCC09 | Renal Cell Carcinoma, Papillary Type 2 | Classic | 13.0 | 3 | T3b N2 Mx |
| 49 | PRCC3 | Renal Cell Carcinoma, Papillary Type 2 | Classic | 2.2 | 3 | T1aNxMx |
| 50 | PRCC2 | Renal Cell Carcinoma, Papillary Type 2 | Classic | 4.3 | 3 | T1bNxMx |
| 51 | MC01 | Low grade neoplasm, favor oncocytoma | Challenging | 3.9 | N/A | N/A |
| 52 | MC02 | Low grade neoplasm, favor oncocytoma | Challenging | 6.7 | N/A | N/A |
| 53 | MC06 | Renal clear cell carcinoma with sarcomatoid differentiation | Challenging |  | 4 | T3a Nx Mx |
| 54 | MC09 | Eosinophilic epithelial tumor morphologically consistent with eosinophilic renal cell carcinoma | Challenging | 16.0 | 3 | T2 Nx Mx |

| n | Sample ID | Diagnosis | Cohort (Morphology) | Size (cm) | Fuhrman Nuclear Grade | TNM Stage |
|---|---|---|---|---|---|---|
| 55 | MC10 | Oncocytic renal neoplasm, favor carcinoma | Challenging | 10.2 | 3 | T2 Nx Mx |
| 56 | MC11 | Renal cell carcinoma, clear cell type with focal granular (eosinophilic) morphology | Challenging | 1.8 | 2 | T1a Nx Mx |
| 57 | MC12 | Eosinophilic variant of clear cell renal carcinoma with papillary features. | Challenging | 4.5 | 3 | T3b N2 Mx |
| 58 | MC13 | Oncocytic renal cell carcinoma, most suggestive of eosinophilic variant of conventional clear cell carcinoma | Challenging | 4.0 | 3 | T3a Nx Mx |
| 59 | MC14 | Chromophobe renal cell carcinoma | Challenging | 6.0 | 3 | T1b Nx Mx |
| 60 | MC15 | Renal cell carcinoma with morphologic features consistent with eosinophilic variant of clear cell carcinoma | Challenging | 1.8 | 3 | T1a Nx Mx |
| 61 | MC16 | Oncocytic renal epithelial neoplasm, favor chromophobe renal cell carcinoma with eosinophilic morphology | Challenging | 4.0 | 3 | T1a Nx Mx |
| 62 | MC18 | Low grade carcinoma with myxoid matrix and spindle and tubular architecture | Challenging | 6.0 | N/A | T1b Nx Mx |
| 63 | MC19 | Renal cell carcinoma with morphologic features of a chromophobe renal cell carcinoma. Multiple other tumors (2 papillary, 2 clear cell) | Challenging | 2.4 | 2 | T1b(5) Nx Mx |
| 64 | MC20 | Eosinophilic renal cell carcinoma | Challenging | 4.3 | 3 | T1b Nx Mx |
| 65 | MC21 | Oncocytic renal epithelial neoplasm | Challenging | 3.0 | 3 | T3a Nx Mx |
| 66 | MC22 | Renal cell carcinoma, unclassified | Challenging | 10.0 | 3 | T2 Nx Mx |
| 67 | MC23 | Eosinophilic renal cell carcinoma | Challenging | 6.0 | 3 | T1b Nx Mx |
| 68 | MC24 | Papillary renal cell carcinoma, type 2 | Challenging | 2.8 | 3 | T1a Nx Mx |
| 69 | MC25 | Oncocytic renal neoplasm, morphologically consistent with an oncocytic variant of conventional clear cell renal cell carcinoma | Challenging | 2.0 | 3 | T1a Nx Mx |
| 70 | MC26 | Clear Cell Carcinoma with features of papillary carcinoma | Challenging | 10.0 | 3 | T3a N0 Mx |
| 71 | MC27 | Renal cell carcinoma not otherwise specified | Challenging | 7.0 | 3 | T3b Nx Mx |
| 72 | MC28 | Renal oncocytoma | Challenging | 5.0 | N/A | N/A |
| 73 | MC29 | Papillary renal cell carcinoma, type II | Challenging | 3.7 | 3 | T1a Nx Mx |
| 74 | MC30 | Renal oncocytoma | Challenging | 21.0 | N/A | N/A |
| 75 | MC31 | Renal epithelial oncocytic neoplasm with features of an oncocytoma | Challenging | 1.5 | 3 | T1a Nx Mx |
