# Supplementary material for: Virtual karyotyping with SNP microarrays reduces uncertainty in the diagnosis of renal epithelial tumors
Source: Diagn Pathol. 2008 Nov 6;3:44. doi: 10.1186/1746-1596-3-44 (PMC2588560; doi:10.1186/1746-1596-3-44)
Supplement: Additional file 2 — Frequency of chromosomal gain/loss identified with SNP arrays in renal cell tumors with classic morphology (n = 50). [file 1746-1596-3-44-S2.pdf]

Supplemental Table 2. Frequency of chromosomal gain/loss identified with SNP arrays in renal cell tumors with classic morphology (n=50).

Chromosomal Arm	Clear Cell RCC		Chromophobe RCC		Papillary RCC		Oncocytoma	
	Loss	Gain	Loss	Gain	Loss	Gain	Loss	Gain
1p	29%	5%	100%		11%		82%	
1q	10%	10%	78%		11%		64%	
2p		14%	100%			11%		
2q	5%	24%	100%			11%		
3p	100%	5%				33%		
3q	14%	14%				44%		
4p					22%			
4q		5%						
5p		52%						9%
5q	10%	76%	11%			11%		9%
6p	19%		100%					
6q	24%		100%					
7p		38%				78%		
7q		33%				78%		
8p	33%		11%		11%			
8q	14%	19%	11%			11%		
9p	19%	10%			11%			
9q	14%	10%			11%			
10p	14%		78%					
10q	24%		78%					
11p	5%							
11q	5%				11%			
12p		19%				44%		
12q		19%				56%		
13q	5%	10%	78%					
14q	43%				11%		18%	9%
15q	5%		11%		11%			
16p		24%				56%		
16q		19%				56%		
17p	5%	5%	100%		11%	78%		
17q	5%	5%	100%			89%		
18p	5%							
18q	5%	5%						
19p		5%	11%			11%		
19q		5%	22%	11%		11%		
20p			22%			22%		
20q		5%	22%			22%		
21q	5%	14%	56%		11%	11%	9%	
22q	10%				22%		18%	
