# Supplementary material for: Virtual karyotyping with SNP microarrays reduces uncertainty in the diagnosis of renal epithelial tumors
Source: Diagn Pathol. 2008 Nov 6;3:44. doi: 10.1186/1746-1596-3-44 (PMC2588560; doi:10.1186/1746-1596-3-44)

Hagenkord et al **Supplemental Table 3 Morphologically Challenging Renal Tumors**

07_0011 (MC01)	Low grade neoplasm, favor oncocytoma
FISH	Not done
SNP	-1, -14, -21
SNP interpretation	Oncocytoma

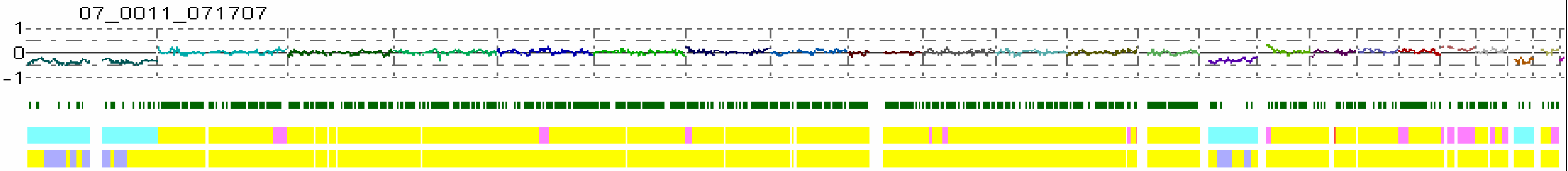

07_0012 (MC02)	Low grade neoplasm, favor oncocytoma
FISH	Not done
SNP	del(10)(p11.23-p14)
SNP interpretation	Oncocytoma

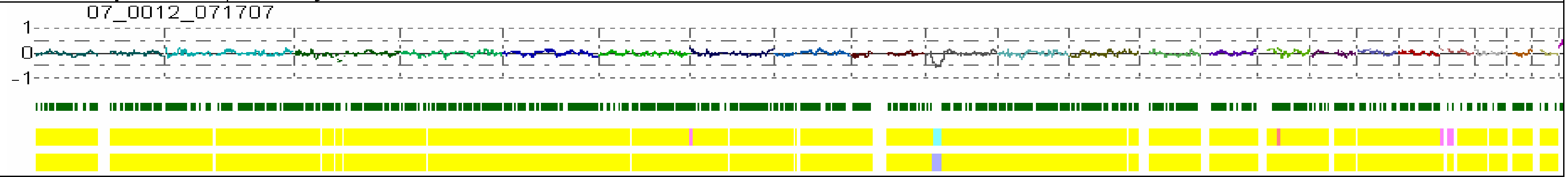

07_0131 (MC09)	Eosinophilic epithelial tumor morphologically consistent with eosinophilic renal cell carcinoma
FISH	-1 (44%), -2 (52%), -7 (38%), and -17 (88%)
SNP	-1, -1, -2, -3, +5, UPD 6, +7, -9, -9, -10p, -10p, del(10)(q24.33-qter), -11, -11, +12, -13,-13, +15(q22.2-qter), +16p, -17p, -17p, 17q, -17q, -18, +19, -21, -21, -22, -22, -22 | tetrasomic?
SNP interpretation	Mixed pattern CRCC / CHRCC = Unclassified

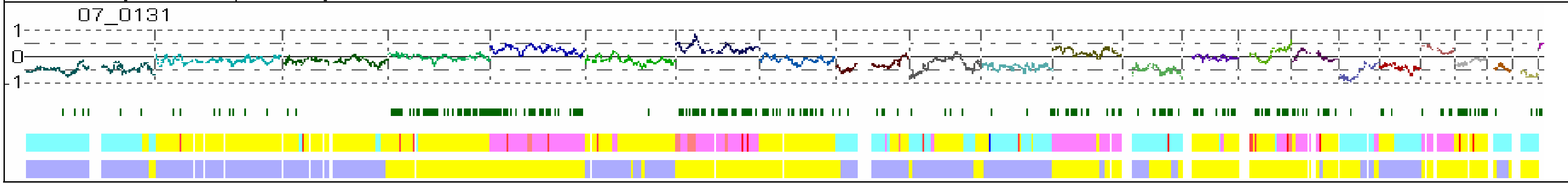

Supplemental Table 3 Morphologically Challenging Renal Tumors

07_0132 (MC10)	Oncocytic renal neoplasm (consensus review: OC)
FISH	-1 (51%), 2 failed, +7 (21%), +7+7 (8%), +17 (30%) and intermediate -17(28%)
SNP	-1, -14
SNP interpretation	Oncocytoma

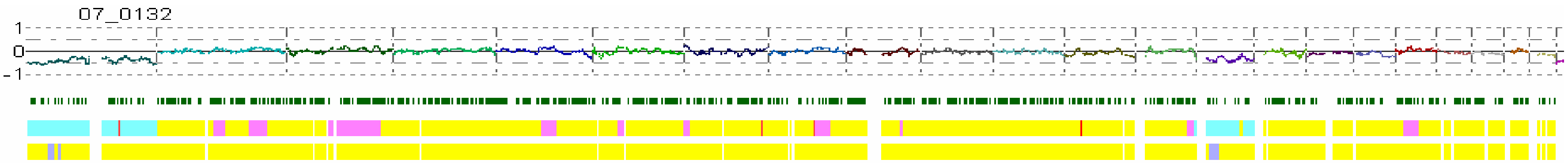

07_0133 (MC11)	Renal cell carcinoma, clear cell type with focal granular morphology (Consensus review: CRCC vs CHRCC)
FISH	CHRCC area: +2 (75%) with intermed -1, -7, -17; CRCC area: -1, -2, -17 (34%, 36%, 30%)
SNP	UPD(3)(p14.1-p13.2)
SNP interpretation	Clear Cell RCC

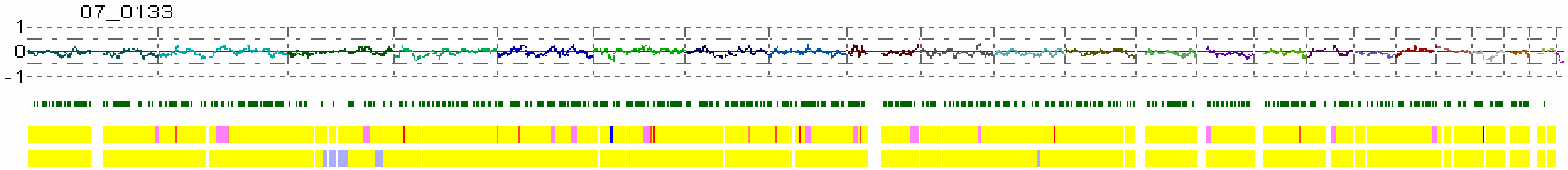

07_0135 (MC13)	Oncocytic RCC most suggestive of eosinophilic variant CRCC
FISH	-1 (97%), -2 (83%), -7 (70%), and -17 (97%)
SNP	UPD(3)(p12.2-p24.1)
SNP interpretation	Clear Cell RCC

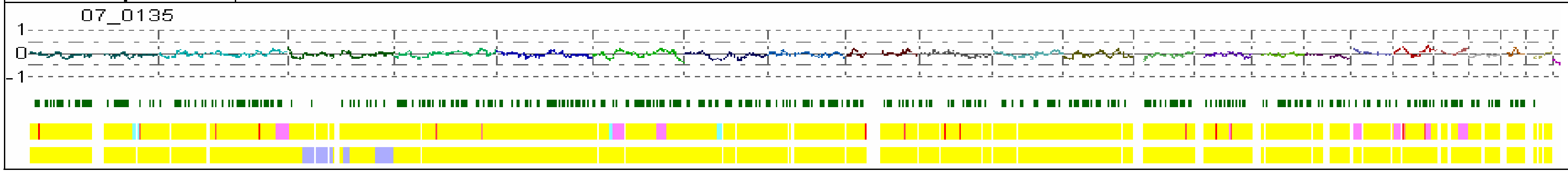

Supplemental Table 3 Morphologically Challenging Renal Tumors

**07_0136 (MC14)**	**Chromophobe renal cell carcinoma**
**FISH**	**-1 (48%), -2 (50%), -7 (56%), and -17 (47%)**
**SNP**	**No detectable chromosomal abnormalities**
**SNP interpretation**	**Oncocytoma**

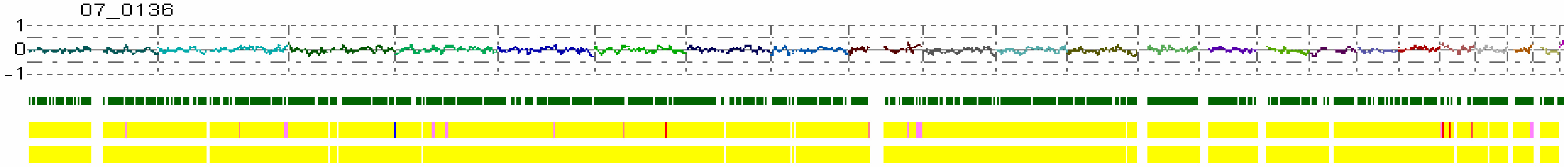

**07_0137 (MC15)**	**Renal cell carcinoma with morphologic features of an eosinophilic variant of clear cell carcinoma**
**FISH**	**-2 (37%), -7 (45%), -17 intermediate (20%)**
**SNP**	**del(1)(p32.3-pter), del(3)(p12.2-pter)**
**SNP interpretation**	**Clear cell RCC**

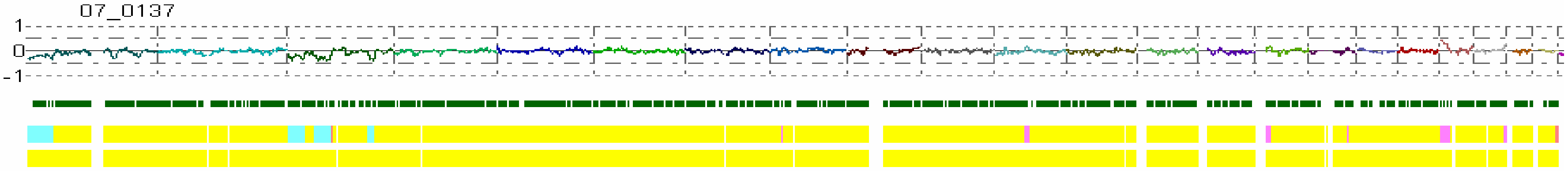

**07_0138 (MC16)**	**Oncocytic renal epithelial neoplasm, favor chromophobe with eosinophilic morphology**
**FISH**	**-1(48%), -2 (40%), -7 (36%), and -17 (42%)**
**SNP**	**-1p, -9q, +12, -18, -21; suspicious for tetrasomy and/or subclones**
**SNP interpretation**	**Oncocytoma (novel)**

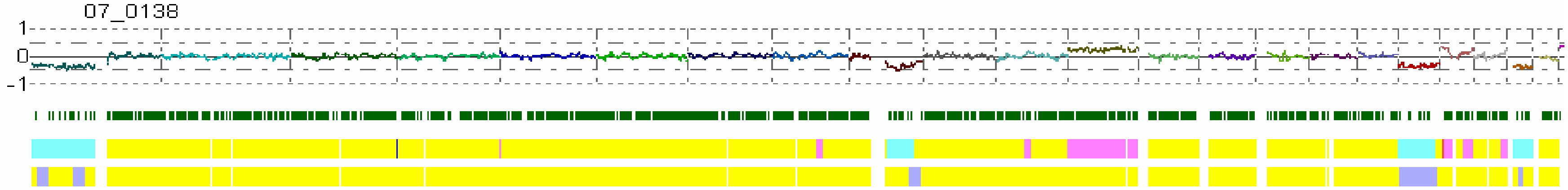

**07_0129 (MC16)**	**Replicate of 07_0138 (same sample extracted and processed 2x)**
**FISH**	
**SNP**	**Not included in statistics because it is a replicate of 07_0138.**
**SNP interpretation**	

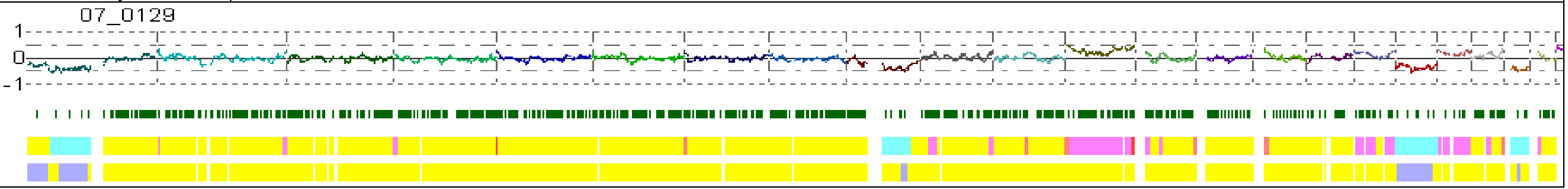

**07_0134 (MC12)**	**Eosinophilic variant of clear cell renal carcinoma with papillary features.**
**FISH**	**-2 (31%), -7 (37%), -17 (97%)**
**SNP**	**del(4)(p15.1-pter), +5(q21.3-qter), +9p(UPD), del(11)(q13.3-qter), +12, +16, del(17p), +17(q21.32-qter), +17(q21.32-qter), +20, -22**
**SNP interpretation**	**Novel, not consistent with clear cell**

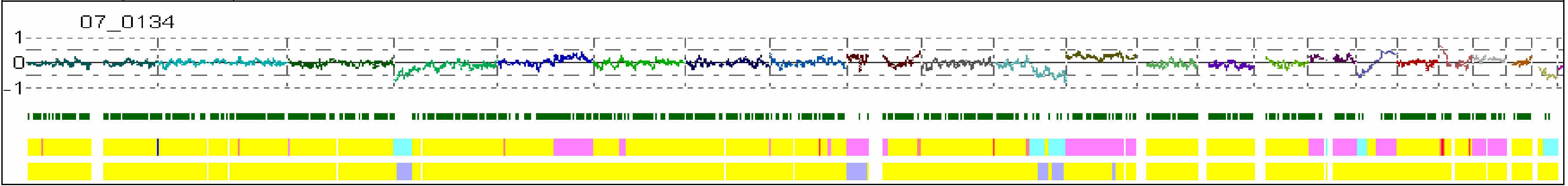

**07_0140 (MC18)**	**Low grade carcinoma with myxoid matrix and spindle and tubular architecture, see comment.**
**FISH**	**-1 (85%), -2 (67%), -7 (37%), and -17 (73%)**
**SNP**	**-1, -4, -6, -8, -9, -13, -14, -15, -17, -22**
**SNP interpretation**	**MTSCC**

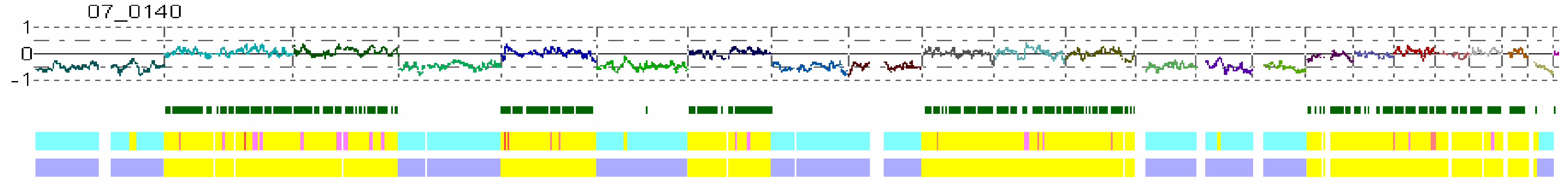

07_0141 (MC19)	Renal cell carcinoma with morphologic features suggestive of chromophobe.
FISH	-1 (38%), -2 (42%), -7 (33%). 17 failed.
SNP	-3p, +3q, +7, del(9)(p13.2-p22.3)
SNP interpretation	Clear cell RCC

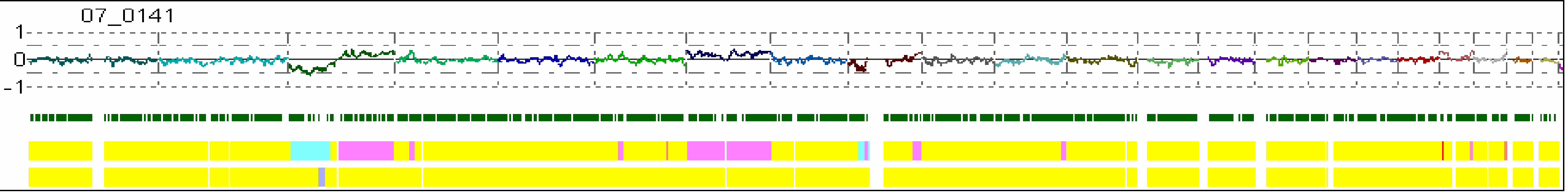

07_0142 (MC20)	Eosinophilic renal cell carcinoma
FISH	Intermed -1 (25%), -2 (52%), -7 (42%), -17 (87%)
SNP	No detectable chromosomal abnormalities
SNP interpretation	Oncocytoma

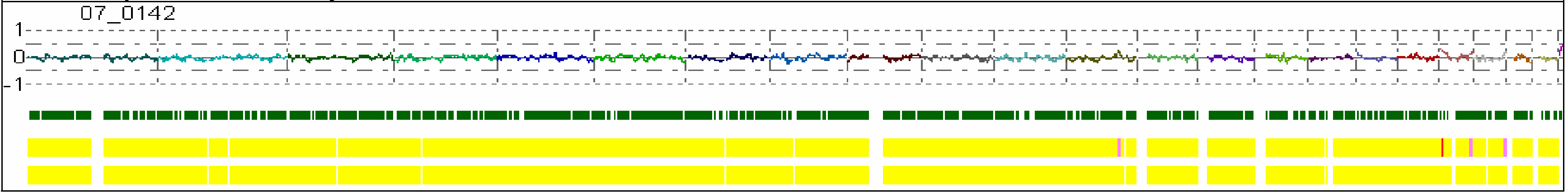

07_0144 (MC21)	Oncocytic renal epithelial neoplasm
FISH	-1 (92%), -17 (37%), +7 (51%)
SNP	-1,+7, +9p, -9q
SNP interpretation	Oncocytoma (novel)

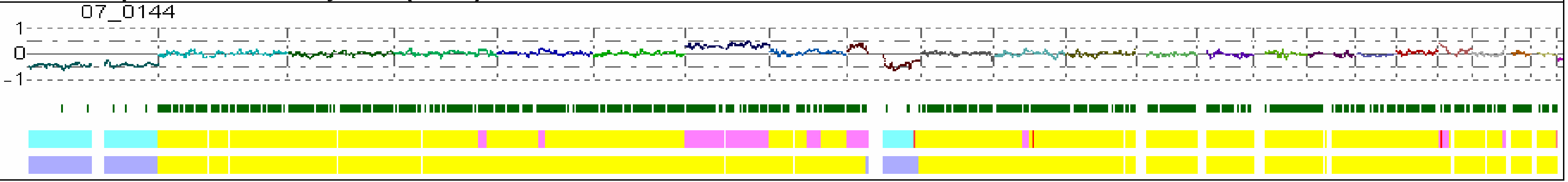

**07_0145 (MC22)**	**Renal cell carcinoma unclassified**
**FISH**	**-1 (45%), 12 (37%), -17 (50%); intermed -7 (22%)**
**SNP**	**+7, +16**
**SNP interpretation**	**Papillary RCC**

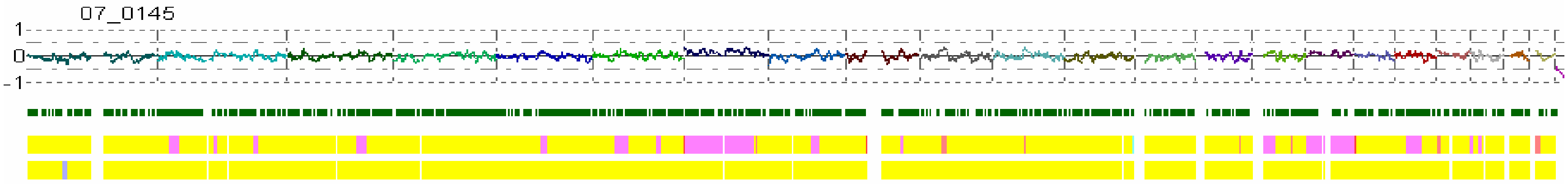

**07_0146 (MC23)**	**Eosinophilic renal cell carcinoma, 6.0cm; addendum supports CHRCC (+parvalbumin)**
**FISH**	**-1 (49%), -2 (53%), -7 (49%), and -17 (74%)**
**SNP**	**No detectable chromosomal abnormalities**
**SNP interpretation**	**Oncocytoma**

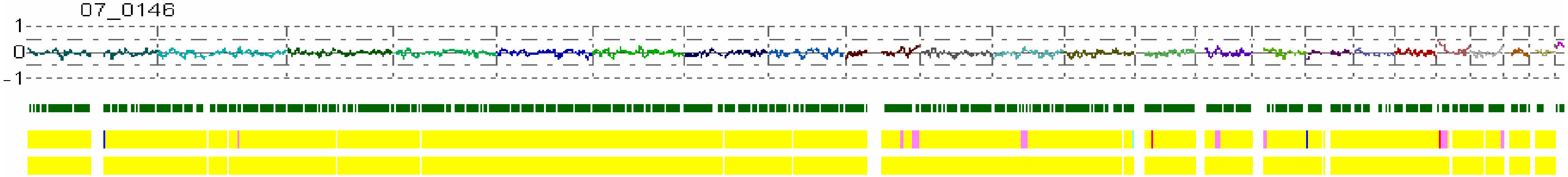

**07_0147 (MC24)**	**Papillary renal cell carcinoma type 2 (Consensus review: PRCC type 1); CHRCC and other PRCCs in same kidney**
**FISH**	**-1 (48%), intermed -2 (23%), -7 (26%)**
**SNP**	**+7, +12, +17**
**SNP interpretation**	**Papillary RCC**

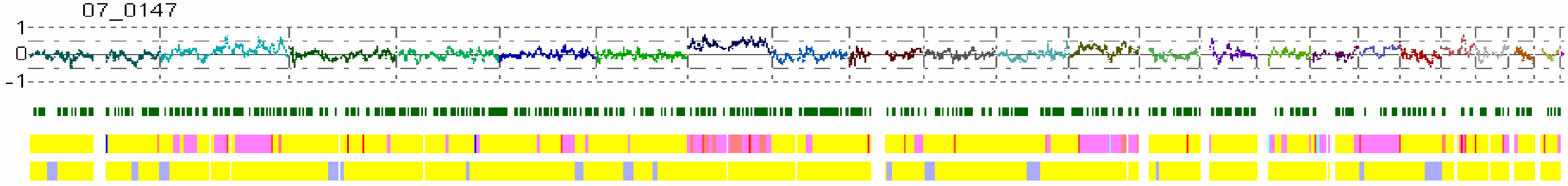

07_0148 (MC25)	Oncocytic renal neoplasm morphologically consistent with oncocytic variant of clear cell carcinoma
FISH	Monosomy 1 (58%), 2 (38%), 7 (48%), and 17 (57%)
SNP	SD too high for clean read. (failed QC)
Comment	No evidence of -3p, probably not CRCC but unsatisfactory for interpretation.

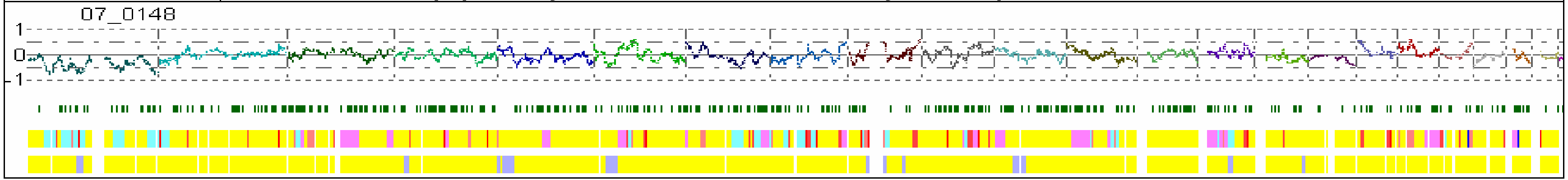

07_0149 (MC26)	Clear cell carcinoma
FISH	1 disomy (82%), 2 intermed monosomy (23%). 7 trisomy (52%), 17 monosomy (43%)
SNP	-3p, unable to interpret other changes
SNP Interpretation	Clear cell carcinoma

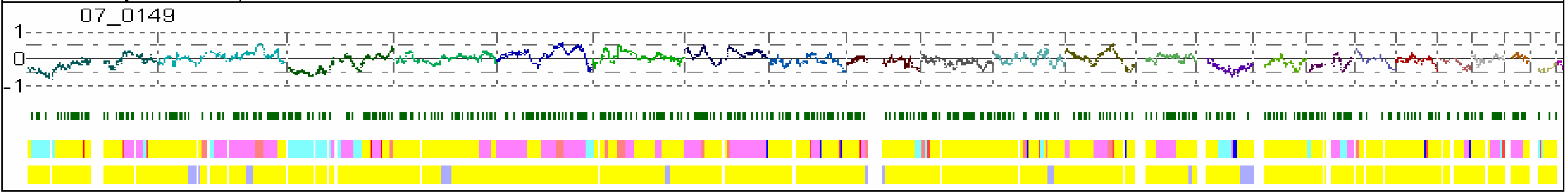

07_0150 (MC27)	Renal cell carcinoma not otherwise specified
FISH	Monosomy 1 (48%), 2 (43%(, 7 (30%), and 17 (58%)
SNP	SD too high for clean read. (failed QC)
Comment	

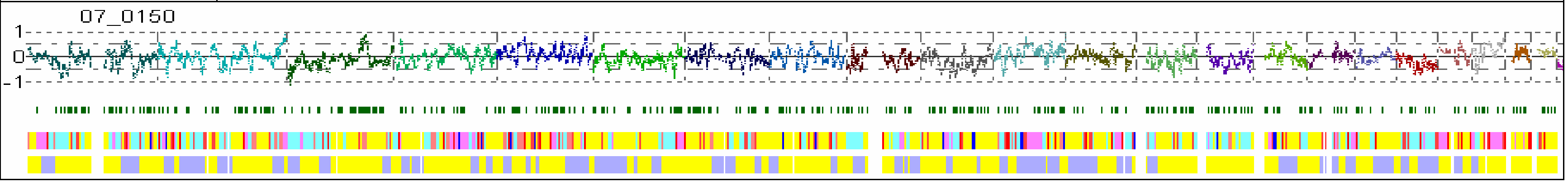

07_0151 (MC28)	Renal oncocytoma
FISH	-1 (37%), -2 (38%), -7 (30%), and -17 (55%)
SNP	No detectable chromosomal abnormalities
SNP interpretation	Oncocytoma

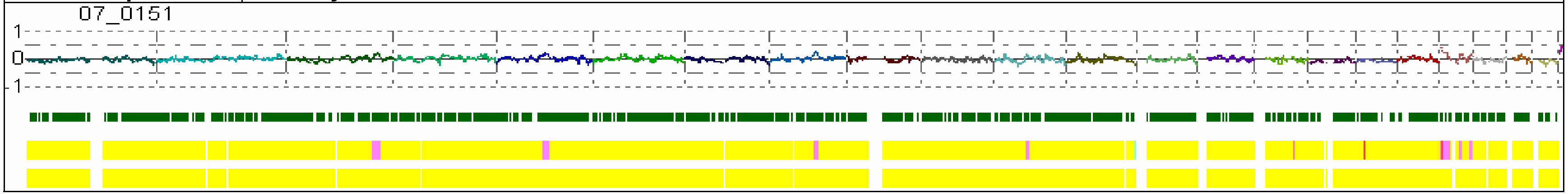

07_0152 (MC29)	Papillary renal cell carcinoma type 2
FISH	-2 intermediate (20%),+7 (30%), +7+7 (18%)
SNP	+7, +16q (signal diluted by normal clone contamination)
SNP interpretation	Papillary RCC

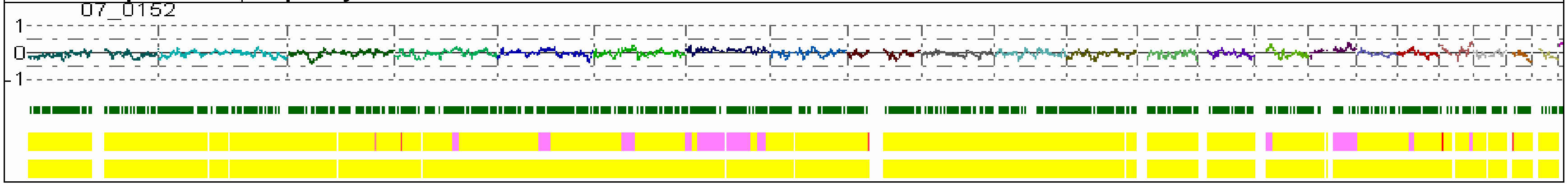

07_0153 (MC30)	Renal oncocytoma
FISH	-1 (41%), 2 failed, -7(34%), -17(65%)
SNP	No detectable chromosomal abnormalities
SNP interpretation	Oncocytoma

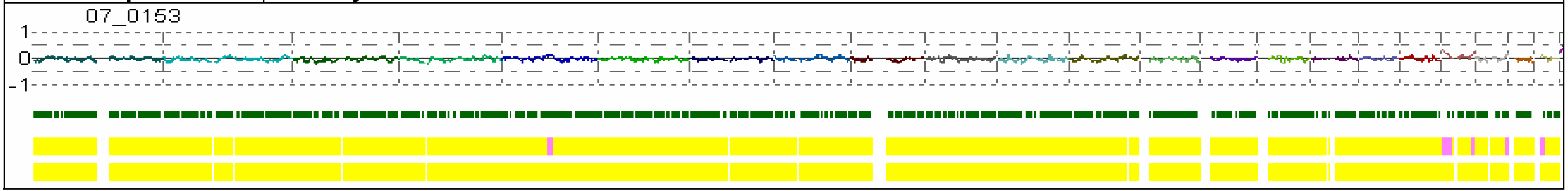

**07_0155 (MC31)**	**Renal epithelial oncocytic neoplasm with features of an oncocytoma**
**FISH**	**-1 (67%), -2 (63%), -7 (42%), and -17 (40%)**
**SNP**	**No detectable chromosomal abnormalities**
**SNP interpretation**	**Oncocytoma**

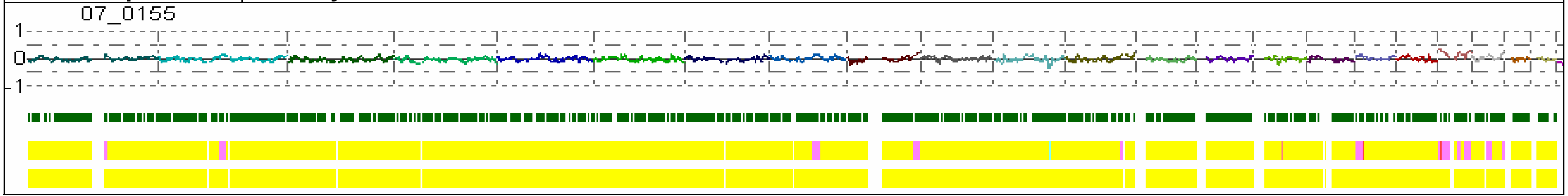

**07_0128 (MC06)**	**Renal clear cell carcinoma with sarcomatoid differentiation**
**FISH**	**Not done**
**SNP**	**SD too high for clean read. (failed QC)**
**Comment**	

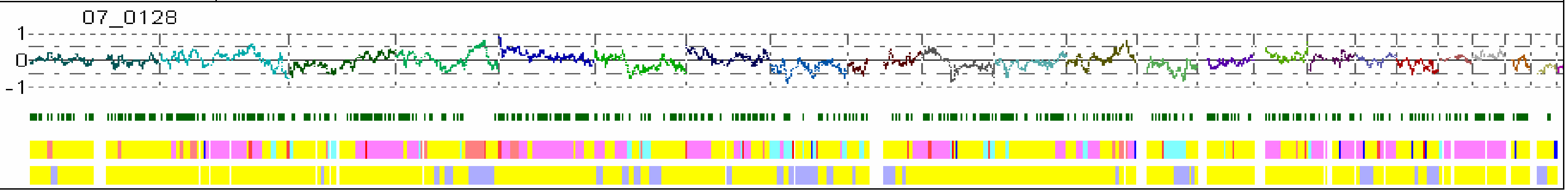

Supplement: Additional file 3 — Virtual karyotypes of morphologically challenging tumors with associated surgical pathology and FISH data. [file 1746-1596-3-44-S3.pdf]
